# Supplementary material for: Cyclical palmitoylation regulates TLR9 signalling and systemic autoimmunity in mice
Source: Nat Commun. 2024 Jan 2;15:1. doi: 10.1038/s41467-023-43650-z (PMC10762000; doi:10.1038/s41467-023-43650-z)
Supplement: Supplementary file 1 — Supplementary Figures [file 41467_2023_43650_MOESM1_ESM.pdf]

Title: Cyclical palmitoylation regulates TLR9 signalling and systemic autoimmunity in mice

Authors: Hai Ni[1,2], Yinuo Wang[3, 4], Kai Yao[2], Ling Wang[2], Jiancheng Huang[2], Yongfang Xiao[2], Hongyao Chen[2], Bo Liu[3, 5 *], Cliff Y. Yang[2, 6 *] and Jijun Zhao[1 *]

Affiliations:

[1]Department of Rheumatology and Immunology, The First Affiliated Hospital, Sun Yat-sen University, Guangzhou, Guangdong, China.

[2]Department of Immunology and Microbiology, Zhongshan School of Medicine, Sun Yat-sen University, Guangzhou, Guangdong, China.

[3]CAS Key Laboratory of Molecular Virology and Immunology, Shanghai Institute of Immunity and Infection, Chinese Academy of Sciences, Shanghai, China.

[4]University of Chinese Academy of Sciences, Beijing, China.

[5]Shanghai Huashen Institute of Microbes and Infections, Shanghai, China

[6]Key Laboratory of Tropical Disease Control (Sun Yat-sen University), Ministry of Education, Guangzhou, China

[*]Corresponding authors:
(B.L.) bliu@ips.ac.cn,
(C.Y.Y.) yangkeli6@mail.sysu.edu.cn
(J.Z.) zhjij@mail.sysu.edu.cn

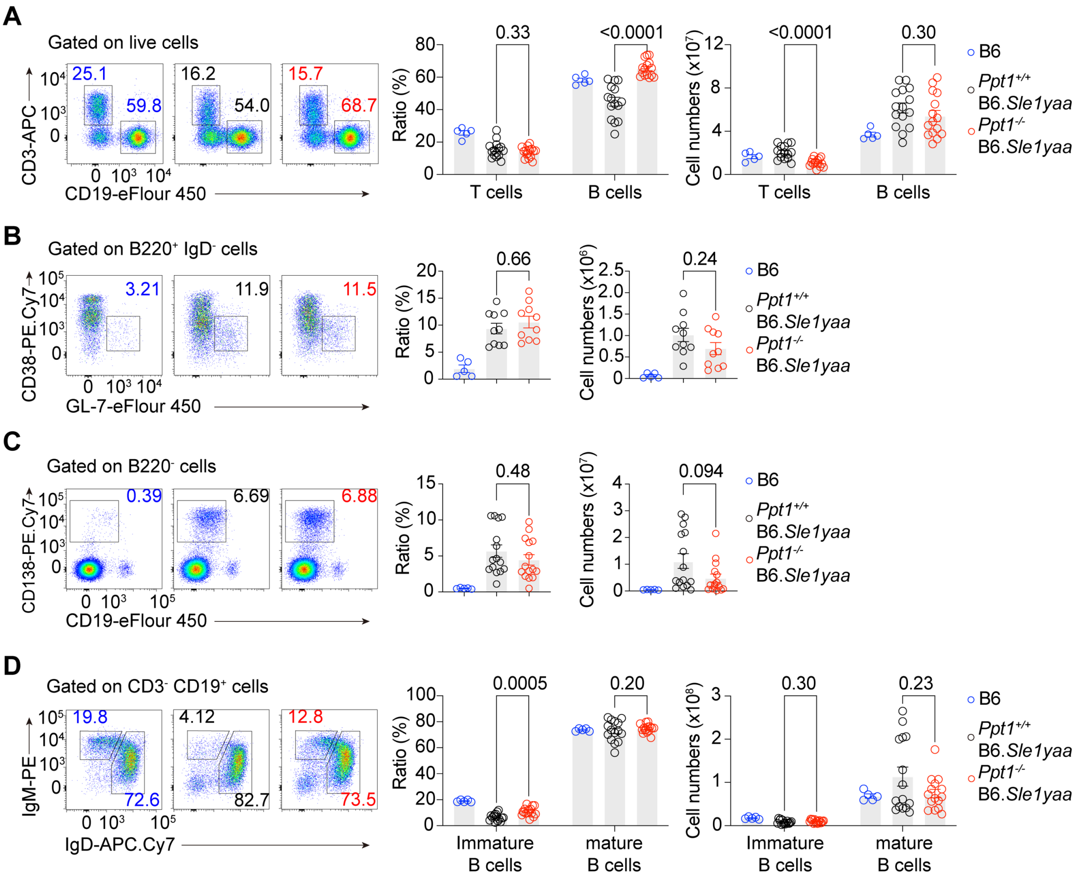

Supplementary Figure 1. The role of PPT1 in SLE pathogenesis.

C57BL/6J (B6), *Ppt1*$^{+/+}$ B6.*Sle1yaa* and *Ppt1*$^{-/-}$ B6.*Sle1yaa* mice were sacrificed at 16 weeks. Immune cell subsets in the spleen were analyzed by FACS. **(A)** Percentage and cell numbers of total T cells (CD3^{+}) and total B cells (CD19^{+}). Representative FACS plots (left), percentages (middle), and cell numbers (right) are shown (n=5 mice in the B6 group; n=15 mice in the *Ppt1*$^{+/+}$ B6.*Sle1yaa* and *Ppt1*$^{-/-}$ B6.*Sle1yaa* groups). **(B)** Percentage and cell numbers of CD38^{-} GL-7^{+} GC B cells in the splenic B-cell population. Representative FACS plots (left), percentages (middle), and cell numbers (right) are shown (n=5 mice in the B6 group; n=10 mice in the *Ppt1*$^{+/+}$ B6.*Sle1yaa* and *Ppt1*$^{-/-}$ B6.*Sle1yaa* groups). **(C)** Percentage and cell numbers of B220^{-} CD19^{-} CD138^{+} plasma B cells. Representative FACS plots (left), percentages (middle), and cell numbers (right) are shown (n=5 mice in the B6 group; n=15 mice in the *Ppt1*$^{+/+}$ B6.*Sle1yaa* and *Ppt1*$^{-/-}$ B6.*Sle1yaa* groups). **(D)** Fractions of IgM^{+} IgD^{-} immature B cells and IgM^{-} IgD^{+} mature B cells gated from splenic CD3^{-} CD19^{+} cell population. Representative FACS plots (left), percentages (middle), and cell numbers (right) are shown (n=5 mice in the B6 group; n=15 mice in the *Ppt1*$^{+/+}$ B6.*Sle1yaa* and *Ppt1*$^{-/-}$ B6.*Sle1yaa* groups). All data are representative of three **(A, C-D)** or two **(B)** independent experiments (mean ± SEM.; P values were calculated by two-way Student's t test).

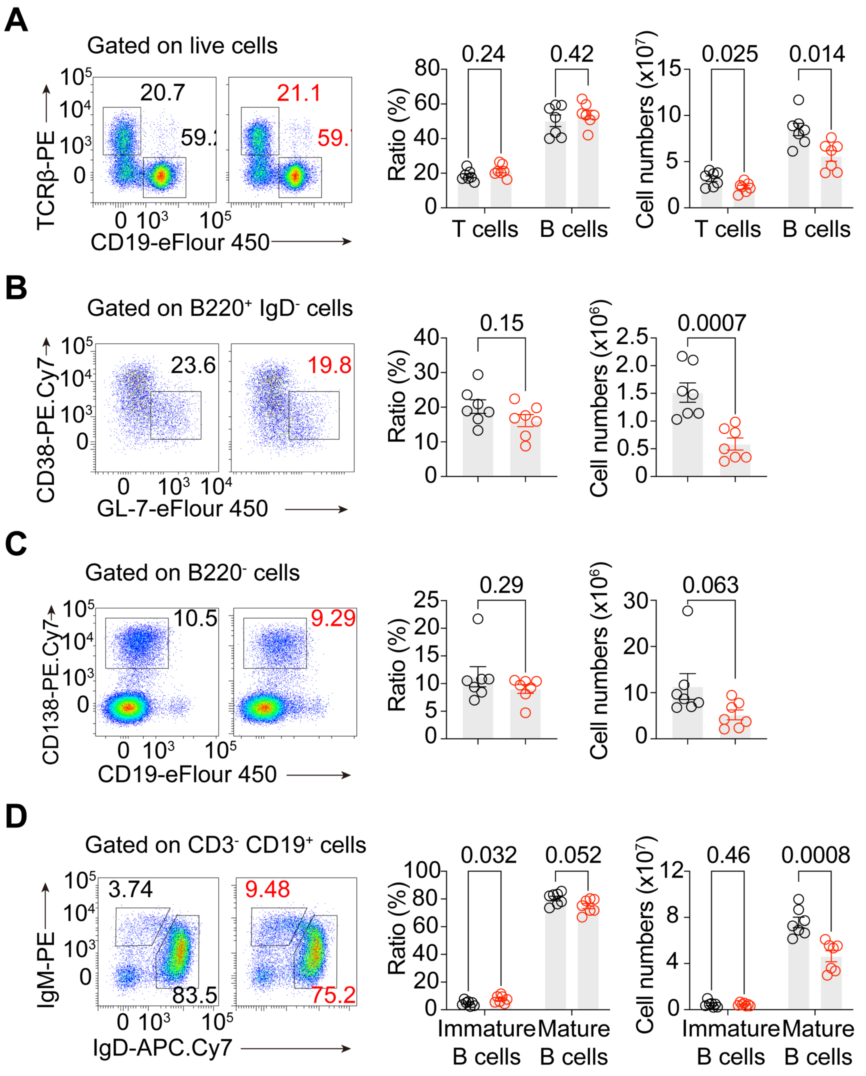

Supplementary Figure 2. **Using PPT1 inhibitors to treat SLE mice.**
B6.*Sle1yaa* mice were treated with DMSO or HDSF for 8 weeks. Immune cell subsets in the spleen were analyzed by FACS (n=7 mice per group for the rest of figure). **(A)** Percentage and cell numbers of total T cells (TCRβ^{+}) and total B cells (CD19^{+}). **(B)** Percentage and cell numbers of CD38^{-} GL-7^{+} GC B cells in the splenic B-cell population. **(C)** Percentage and cell numbers of B220^{-} CD19^{-} CD138^{+} plasma B cells. **(D)** Percentage and cell numbers of IgM^{+} IgD^{-} immature B cells and IgM^{-} IgD^{+} mature B cells gated from the splenic CD3^{-} CD19^{+} cell population. All data are representative of three independent experiments (mean ± SEM.; P values were calculated by two-way Student's t test).

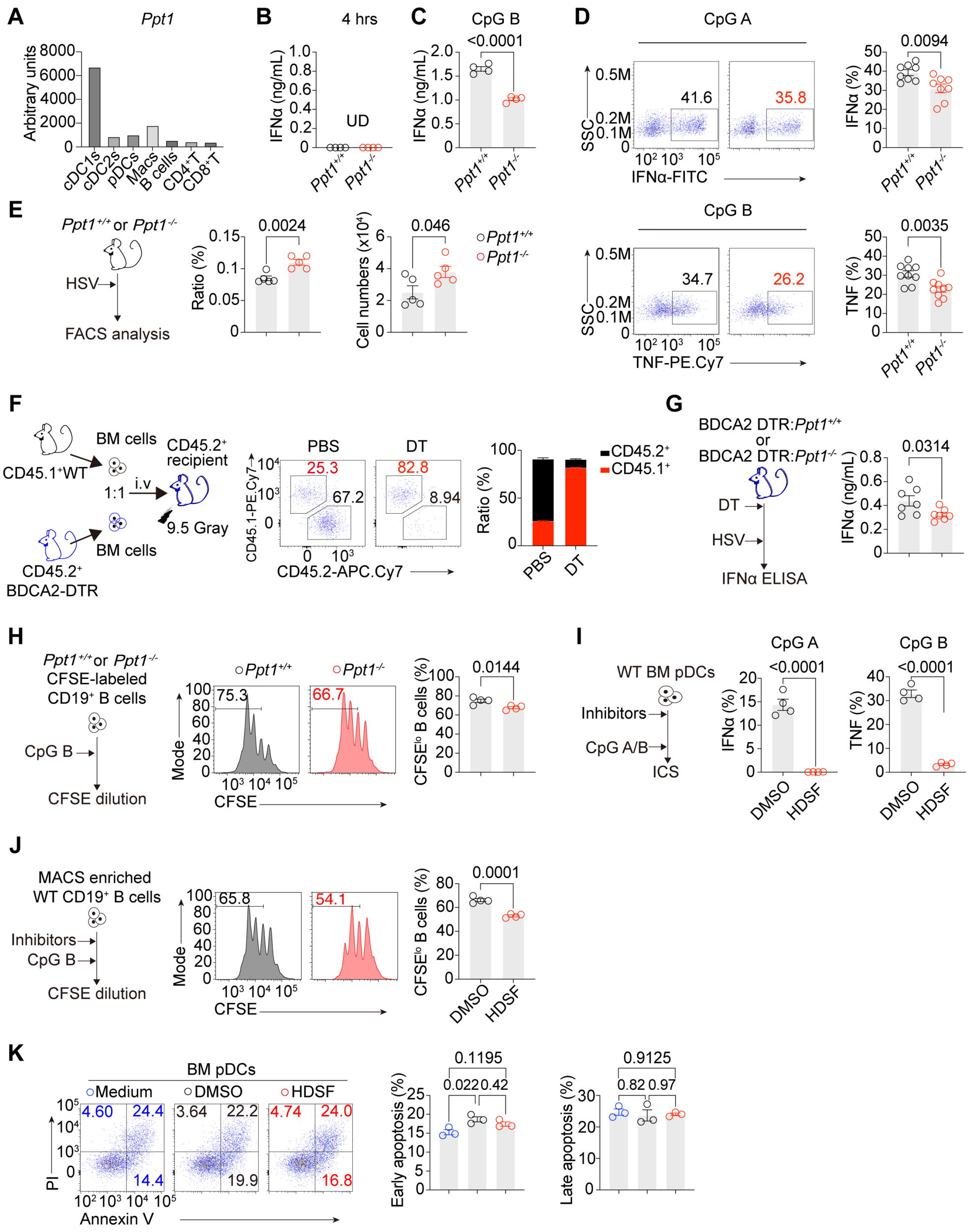

Supplementary Figure 3. Construction of *Ppt1*$^{+/+}$ or *Ppt1*$^{-/-}$ chimeras.

(A) Expression of *Ppt1* (in arbitrary unit) in the indicated populations reanalyzed from ImmGen gene microarray. **(B-C)** Sorted *Ppt1*$^{+/+}$ or *Ppt1*$^{-/-}$ BM pDCs were treated with CpG A for 4 hours **(B)** or CpG B overnight **(C)**, IFNα production was measured by ELISA (n = 4 mice per group), UD: Undetectable. **(D)** *Ppt1*$^{+/+}$ or *Ppt1*$^{-/-}$ BM pDCs were treated with CpG A (left) or CpG B (right), and IFNα (left) and TNF (right) production was measured by intracellular staining (n = 8 mice per group for CpG A, n = 9 mice per group for CpG). **(E)** *Ppt1*$^{+/+}$ and *Ppt1*$^{-/-}$ mice were infected with HSV (left). Spleen pDCs ratio (middle) and numbers (right) for HSV are shown (n=5 mice for HSV). **(F)** Generation of BDCA2-DTR:*Ppt1*$^{+/+}$ and BDCA2-DTR:*Ppt1*$^{-/-}$ mixed chimeras. An equal number of bone marrow cells from CD45.2^{+} BDCA2-DTR mice and CD45.1^{+} WT mice were injected into lethally irradiated CD45.2^{+} mice. After 8 weeks of restitution, the deletion rate of CD45.2^{+} pDCs in the spleen after DT treatment (right), as measured by flow cytometry (gated according to live B220^{+} CD11c^{int} SiglecH^{+} Bst-2^{+} cells in the spleen, n = 3 mice per group). **(G)** BDCA2-DTR:*Ppt1*$^{+/+}$ and *Ppt1*$^{-/-}$ mixed chimeras were injected with DT before infection (left). Serum IFNα ELISAs for HSV are shown (n=7 mice for HSV). **(H)** *Ppt1*$^{+/+}$ or *Ppt1*$^{-/-}$ B cell proliferation was measured after stimulation for 3 days with CpG B. (n=4 mice per group). **(I)** DMSO or HDSF pretreated WT BM pDCs were treated with CpG A or CpG B (left), and IFNα (middle) and TNF (right) production was measured by intracellular staining (n=4 mice per group). **(J)** DMSO or HDSF treated WT B cell proliferation was measured after stimulation for 3 days with CpG B. (n=4 mice per group). **(K)** BM pDCs apoptosis were analyzed by Annexin V and PI staining (n = 3 mice per group). The data are representative of three independent experiments **(B-F, H-K)**, two **(G)** experiments (mean ± SEM.; P values were calculated by two-way Student's t test), or one independent experiment **(F)**.

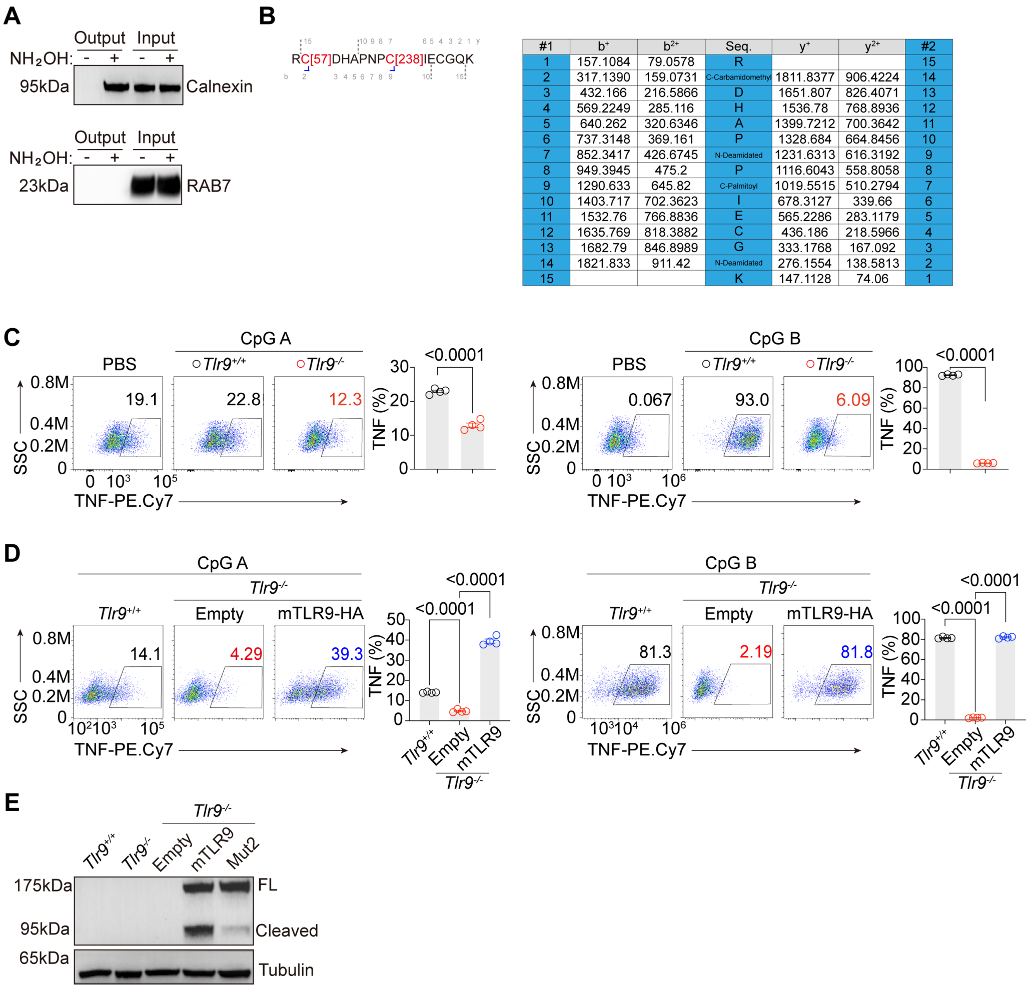

#1	b⁺	b²⁺	Seq.	y⁺	y²⁺	#2
1	157.1084	79.0578	R			15
2	317.1390	159.0731	C-Carbamidomethyl	1811.8377	906.4224	14
3	432.166	216.5866	D	1651.807	826.4071	13
4	569.2249	285.116	H	1536.78	768.8936	12
5	640.262	320.6346	A	1399.7212	700.3642	11
6	737.3148	369.161	P	1328.684	664.8456	10
7	852.3417	426.6745	N-Deamidated	1231.6313	616.3192	9
8	949.3945	475.2	P	1116.6043	558.8058	8
9	1290.633	645.82	C-Palmitoyl	1019.5515	510.2794	7
10	1403.717	702.3623	I	678.3127	339.66	6
11	1532.76	766.8836	E	565.2286	283.1179	5
12	1635.769	818.3882	C	436.186	218.5966	4
13	1682.79	846.8989	G	333.1768	167.092	3
14	1821.833	911.42	N-Deamidated	276.1554	138.5813	2
15			K	147.1128	74.06	1

Supplementary Figure 4. Construction of the *Tlr9*$^{-/-}$ *Tlr9*^{Mut2} cell line.

(A) Controls for the ABE assays. Calnexin (left) was used as the positive control, and RAB7 (right) was used as the negative control. **(B)** The value of b ion and y ion for selected peptide spectra for mTLR9 C258 and C265. **(C)** TNF production in *Tlr9*$^{-/-}$ RAW264.7 cells. Cells were treated with CpG A (left) or CpG B (right) and then subjected to intracellular cytokine staining (n = 4 replicates from 3 independent experiments). **(D)** TNF production in *Tlr9*$^{-/-}$ RAW264.7 cells overexpressing TLR9-HA. The cells were treated with CpG A (left) or CpG B (right) and then subjected intracellular cytokine staining (n = 4 replicates from 3 independent experiments). **(E)** Protein expression of mTLR9 or mTLR9 C258A+C265A (*Tlr9*^{Mut2}) mutants (Mut2) in the *Tlr9*$^{-/-}$ RAW 264.7 cell line. The data are representative of at least five **(A)**, three **(C-E)** independent experiment (mean ± SEM. P values were calculated by two-way Student's t test), **(B)** were obtained from one mass spectrometry run.

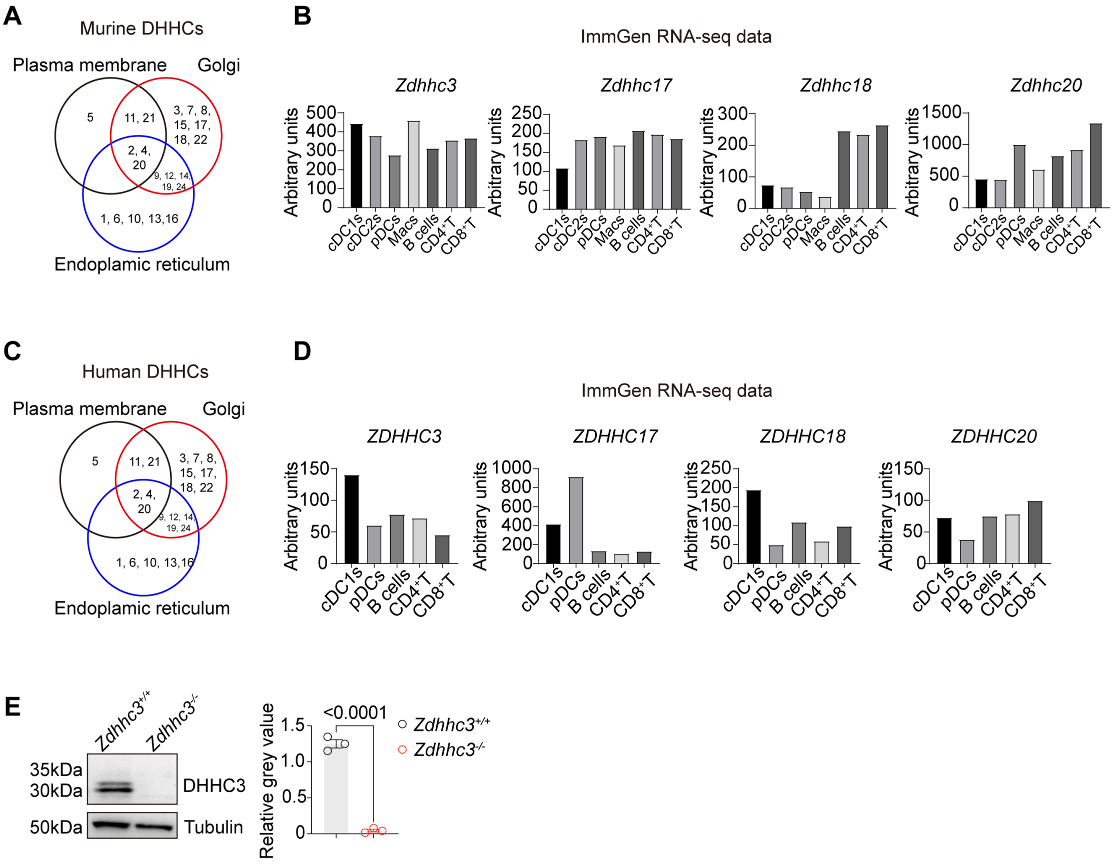

Supplementary Figure 5. Ubiquitous expression of Golgi DHHC proteins.

(**A**) Venn diagrams showing the intracellular locations of all murine DHHCs. **(B)** Murine *Zdhhc* expression in immune cells. RNA-seq datasets from ImmGen were downloaded, and the results are shown for the indicated populations using arbitrary units. (**C**) Venn diagrams showing the intracellular locations of all human DHHCs. **(D)** Human *ZDHHC* expression in immune cells. RNA-seq datasets from ImmGen were downloaded, and the results are shown for the indicated populations using arbitrary units. **(E)** Validation of DHHC3-deficient RAW264.7 cell lines by Western blotting (n=3 replicates). Data is representative of three independent experiments (mean ± SEM. P values were calculated by two-way Student's t test).

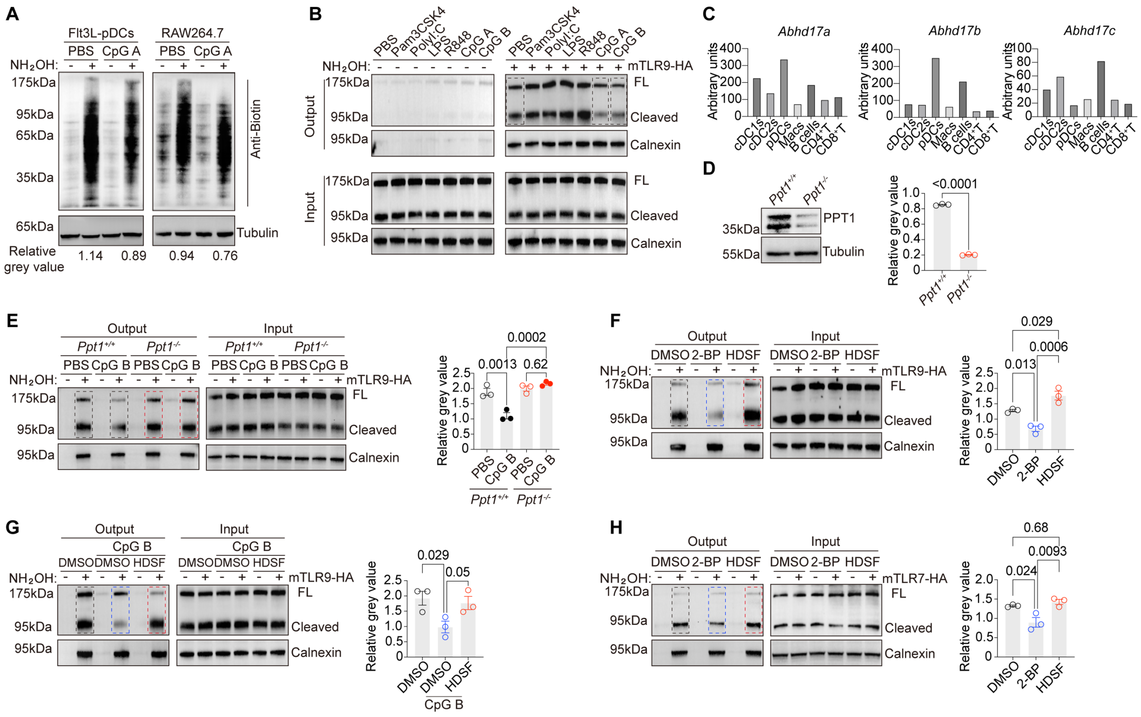

Supplementary Figure 6. Identification of PPT1 as a depalmitoylating enzyme. **(A)** An ABE assay was performed to measure the total S-palmitoylation level in Flt3L-pDCs (left) and RAW264.7 cells (right) treated with CpG A. **(B)** mTLR9-HA was retrovirally transduced into RAW264.7 cells. The cells were treated with the indicated TLR agonists for 4 hours and then subjected to an ABE assay. A representative SDS–PAGE gel is shown. **(C)** Expression of *Abhd17a, Abhd17b*, *Abhd17c* (in arbitrary unit) in the indicated populations reanalyzed from ImmGen gene microarray. **(D)** Validation of PPT1-deficient RAW264.7 cell lines by Western blotting (n=3 replicates). **(E)** TLR9 palmitoylation in PPT1-deficient cell lines. PPT1 in RAW264.7 cells was knocked out by CRISPR/Cas9. The cells were then retrovirally transduced with mTLR9-HA. After CpG B activation for 4 hours, ABE assays were performed. A representative SDS–PAGE gel is shown on the left. The relative palmitoylation levels of mTLR9 shown on the right were quantified by ImageJ and calculated as a percentage of the WT PPT1 signal (n=3 replicates). **(F)** mTLR9 palmitoylation after inhibitor treatment. RAW264.7 cells overexpressing mTLR9-HA were incubated with DMSO, 2-BP or HDSF, and then, ABE assays were performed (n=3 replicates). **(G)** TLR9 palmitoylation status after PPT1 inhibition. mTLR9-HA was retrovirally transduced into RAW264.7 cells. The cells were pretreated with DMSO or HDSF overnight before the CpG B was added. An ABE assay was performed after 4 hours. A representative SDS–PAGE gel is shown on the left. mTLR9 S-palmitoylation was quantified and presented as the ratio of mTLR9 output to calnexin output on the right (n=3 replicates). **(H)** mTLR7 palmitoylation after inhibitor treatment. RAW264.7 cells overexpressing mTLR9-HA were incubated with DMSO, 2-BP or HDSF, and then, ABE assays were performed (n=3 replicates). Data are pooled from three **(B, D-H)** or two **(A)** independent experiments (mean ± SEM. P values were calculated by two-way Student's t test).

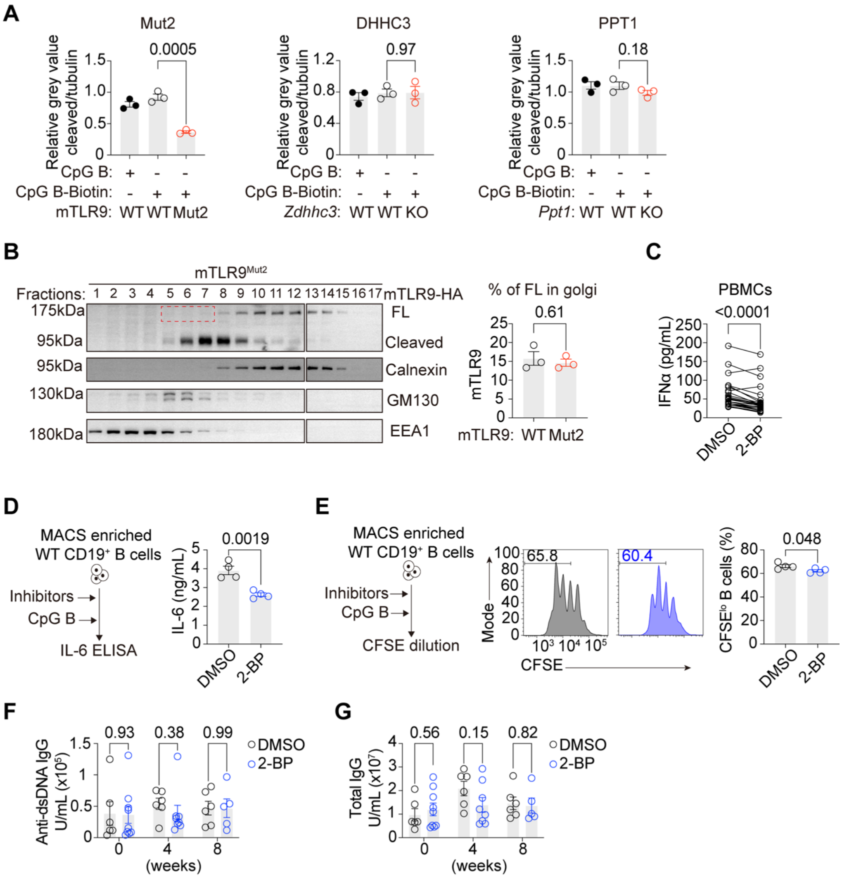

Supplementary Figure 7. **The role of palmitoylation inhibitors in SLE pathogenesis.** **(A)** Quantification of the cleaved TLR9 in mTLR9^{Mut2} *Zdhhc3*$^{-/-}$ or *Ppt1*$^{-/-}$ input before pull down were shown. **(B)** Cell fractionation of *Tlr9*$^{-/-}$ RAW264.7 cells transduced with mTLR9-HA or TLR9 C258A+C265A (mTLR9^{Mut2}) showing the distributions of TLR9 in the indicated organelle markers. A representative blot (left) and the ratio of the cleaved mTLR9 in endosome (right, quantified as the ratio of the boxed fractions to all fractions, n=3 replicates). **(C)** PBMCs of SLE patients were treated with DMSO or 2-BP overnight. After CpG A stimulation, IFNα levels was evaluated by ELISAs (n= 26 individuals for 2-BP/DMSO). **(D)** CD19$^+$ B cells enriched from B6 mice spleen were treated with 2-BP (left). After CpG B stimulation, IL-6 levels were evaluated by ELISAs (n = 4 mice per group). **(E)** CD19$^+$ B cells enriched from B6 mice spleen were labelled with CFSE. B cells proliferation was measured after 2-BP and CpG B stimulation for 3 days. **(F-G)** B6.*Sle1yaa* mice were treated with DMSO or 2-BP for 8 weeks. Serum was collected from each mouse group at the indicated timepoints, and anti-DNA antibody (**F**) and total IgG (**G**) levels were measured by ELISAs (n = 5 mice in the DMSO treatment group; n = 9 mice in the 2-BP treatment group). All data are representative of three independent experiments (mean ± SEM. P values were calculated by two-way Student's t test), except for those presented in **C**, which were pooled from six independent experiments (P values were calculated by two-tailed Wilcoxon matched-pair signed rank test).

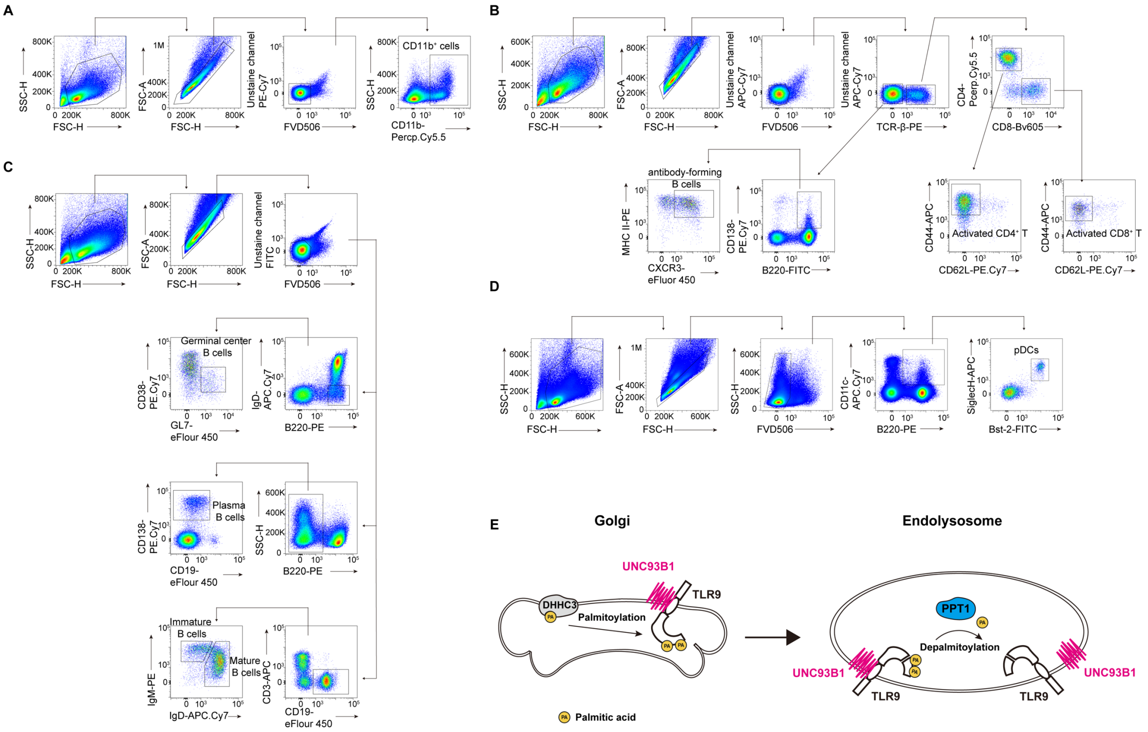

Supplementary Figure 8. **Flow cytometry gating strategies.**

(A) Gating strategy to identify CD11b$^+$ cells presented on Fig. 1G; Fig. 2H. **(B)** Gating strategy to identify activated CD4$^+$ T cells presented on Fig. 1H; Fig. 2I and activated CD8$^+$ T cells presented on Fig. 1I; Fig. 2J. Gating strategy to identify antibody-forming B cells presented on Fig. 1J; Fig. 2K. **(C)** Gating strategy to identify germinal center B cells presented on Supplementary Fig1. B; Supplementary Fig2. B. Gating strategy to identify plasma B cells presented on Supplementary Fig1. C; Supplementary Fig2. C. Gating strategy to identify immature and mature B cells presented on Supplementary Fig1. D; Supplementary Fig2. D. **(D)** Gating strategy to identify pDCs cells was used in Fig. 3C; Fig. 3F; Supplementary Fig3. B-F, I, K. **(E)** A schematic of the working model.
