# Supplementary material for: Cyclical palmitoylation regulates TLR9 signalling and systemic autoimmunity in mice
Source: Nat Commun. 2024 Jan 2;15:1. doi: 10.1038/s41467-023-43650-z (PMC10762000; doi:10.1038/s41467-023-43650-z)
Supplement: Supplementary file 3 — Reporting Summary [file 41467_2023_43650_MOESM3_ESM.pdf]

nature portfolio

Corresponding author(s): Jijun Zhao

Last updated by author(s): Oct 29, 2023

Reporting Summary

Nature Portfolio wishes to improve the reproducibility of the work that we publish. This form provides structure for consistency and transparency in reporting. For further information on Nature Portfolio policies, see our Editorial Policies and the Editorial Policy Checklist.

Statistics

For all statistical analyses, confirm that the following items are present in the figure legend, table legend, main text, or Methods section.

n/a	Confirmed	
☐	☒	The exact sample size (*n*) for each experimental group/condition, given as a discrete number and unit of measurement
☐	☒	A statement on whether measurements were taken from distinct samples or whether the same sample was measured repeatedly
☐	☒	The statistical test(s) used AND whether they are one- or two-sided<br>*Only common tests should be described solely by name; describe more complex techniques in the Methods section.*
☐	☒	A description of all covariates tested
☐	☒	A description of any assumptions or corrections, such as tests of normality and adjustment for multiple comparisons
☐	☒	A full description of the statistical parameters including central tendency (e.g. means) or other basic estimates (e.g. regression coefficient) AND variation (e.g. standard deviation) or associated estimates of uncertainty (e.g. confidence intervals)
☐	☒	For null hypothesis testing, the test statistic (e.g. *F*, *t*, *r*) with confidence intervals, effect sizes, degrees of freedom and *P* value noted<br>*Give P values as exact values whenever suitable.*
☒	☐	For Bayesian analysis, information on the choice of priors and Markov chain Monte Carlo settings
☒	☐	For hierarchical and complex designs, identification of the appropriate level for tests and full reporting of outcomes
☒	☐	Estimates of effect sizes (e.g. Cohen's *d*, Pearson's *r*), indicating how they were calculated

Our web collection on statistics for biologists contains articles on many of the points above.

Software and code

Policy information about availability of computer code

Data collection	Flow cytometric data: Beckman Coulter CytoFLEX<br>mRNA expression data: StepOne Plus (Life Sciences)<br>Immunoblot images: ChemiDoc Touch (Bio-Rad).<br>Immunofluorescence data: Keyence fluorescence microscope (BZ-X710).<br>Mass spectrometry data: nanoflow UPLC system (Thermo Fisher Scientific), Q Exactive Hybrid Quadrupole-Orbitrap mass spectrometer (Thermo Fisher Scientific)
Data analysis	Flow cytometric data:FlowJo vX;<br>Statistical data: Prism v8<br>Immunoblot quantification: ImageJ (Fiji Version: 2.1.0/1.53c)<br>Mass spectrometry data were analyzed using Byonic (Version 4.3).

For manuscripts utilizing custom algorithms or software that are central to the research but not yet described in published literature, software must be made available to editors and reviewers. We strongly encourage code deposition in a community repository (e.g. GitHub). See the Nature Portfolio guidelines for submitting code & software for further information.

Data

Policy information about availability of data

All manuscripts must include a data availability statement. This statement should provide the following information, where applicable:

- Accession codes, unique identifiers, or web links for publicly available datasets
- A description of any restrictions on data availability
- For clinical datasets or third party data, please ensure that the statement adheres to our policy

No novel datasets or reagents are generated in this study.
The RNA-seq datasets that support the findings of this study are publicly available from The Immunological Genome Project (http://rstats.immgen.org/Skyline_microarray/skyline.html), and these data were reanalyzed in the study.

Research involving human participants, their data, or biological material

Policy information about studies with human participants or human data. See also policy information about sex, gender (identity/presentation), and sexual orientation and race, ethnicity and racism.

Reporting on sex and gender	There is no sex and gender potential bias in this study.
Reporting on race, ethnicity, or other socially relevant groupings	There is no race, ethnicity, or other socially relevant groupings were used in this study.
Population characteristics	No covariate-relevant population characteristics were used.
Recruitment	Informed consent was obtained from all subjects. All patients met the American College of Rheumatology classification criteria for systemic lupus erythematosus. Patients with concurrent infection, malignancy or other autoimmune diseases were excluded from the study. There was no exclusion criterion for healthy volunteers. Peripheral blood samples were collected from patients or healthy donors. There is no potential bias in this study as there is no different treatments or groups, all samples are used in the analysis.
Ethics oversight	Approved by the Ethics Committee of The First Affiliated Hospital, Sun Yat-sen University (Approval No. 2022-436)

Note that full information on the approval of the study protocol must also be provided in the manuscript.

Field-specific reporting

Please select the one below that is the best fit for your research. If you are not sure, read the appropriate sections before making your selection.

☒ Life sciences ☐ Behavioural & social sciences ☐ Ecological, evolutionary & environmental sciences

For a reference copy of the document with all sections, see nature.com/documents/nr-reporting-summary-flat.pdf

Life sciences study design

All studies must disclose on these points even when the disclosure is negative.

Sample size	No statistical methods were used to predetermine sample size. Sample size were chosen based on the maximum number of mice available at the moment of experiments. Since the mice are on identical genetic backgrounds, a minimum of 3 individual mice were commonly recognized in the field to sufficiently detect differences between genotypes or conditions. Sample size was determined based on standards for experimental cell lines, attempting to have a minimum of n=3 biological replicates with sufficient reproducibility.
Data exclusions	No data exclusions in all experiments.
Replication	Experiments were replicated successfully for least twice as described throughout the paper and in the Methods.
Randomization	Mice of similar ages and sex were used for all the experiments reported. Samples were randomly assigned.
Blinding	For mice kidney H&E analysis and immunofluorescence analysis, glomerular size, immunofluorescence intensity of IgG and C3 were performed blinded, without knowledge of mice genotype. For other experiments, the investigators were not blinded to the identities of the samples because treatments and data collection were performed by the same people. During data analysis, investigators were not blinded to group allocation, as this is performed by the same people. The reason for these unblindings is due to a lack of personnel and resources, as investigators have to collect and analyze data by themselves. For human studies, there was no blinding in this study as we did not have different treatment/experimental groups and therefore was not relevant.

Reporting for specific materials, systems and methods

We require information from authors about some types of materials, experimental systems and methods used in many studies. Here, indicate whether each material, system or method listed is relevant to your study. If you are not sure if a list item applies to your research, read the appropriate section before selecting a response.

Materials & experimental systems

n/a	Involved in the study
☐	☒ Antibodies
☐	☒ Eukaryotic cell lines
☒	☐ Palaeontology and archaeology
☐	☒ Animals and other organisms
☒	☐ Clinical data
☒	☐ Dual use research of concern
☒	☐ Plants

Methods

n/a	Involved in the study
☒	☐ ChIP-seq
☐	☒ Flow cytometry
☒	☐ MRI-based neuroimaging

Antibodies

Antibodies used

For magnetic activated cell enrichment,
Company: Invitrogen IgG Catalog #: 13-4013-85
Company: Invitrogen IgD clone: 11-26 Catalog #: 13-5993-82
Company: Invitrogen CD93 clone: AA4.1 Catalog #: 13-5892-82
Company: Invitrogen CD5 clone: 53-7.3 Catalog #: 13-0051-82
Company: Invitrogen CD49b clone: DX5 Catalog #: 13-5971-85
Company: Invitrogen TCR β clone: H57-597 Catalog #: 13-5961-85
Company: Invitrogen CD11b clone: M1170 Catalog #: 13-0112-82
Company: Invitrogen CD24 clone: M1/69 Catalog #: 13-0242-82
Company: Invitrogen F4/80 clone: BM8 Catalog #: 13-4801-81
Company: BioLegend Ly6G/Ly6C (Gr-1) clone: R86-8C5 Catalog #: 108404
Company: BioLegend CD41 clone: MWReg30 Catalog #: 133930
Company: TONBO CD19 clone: 1D3 Catalog #: 30-0193-U500
Company: TONBO TER-119 clone: TER-119 Catalog #: 30-5921-U500
Company: TONBO CD3 clone: 145-2C11 Catalog #: 30-0031-U500

For flow cytometry ,
Company: Invitrogen CD19 eFluor 450 Clone: 1D3 Catalog #: 48-0193-82
Company: Invitrogen CD3e APC clone: 17A2 Catalog #: 17-0032-82
Company: Invitrogen CD11b PerCP-Cyanine5.5 clone: M1/70 Catalog #: 45-0112-82
Company: Invitrogen IgD APC-Cyanine7 clone: 11-26c Catalog #: 47-5993-82
Company: Invitrogen IgM PE clone: II/41 Catalog #: 12-5790-82
Company: Invitrogen B220 FITC clone: RA3-6B2 Catalog #: 11-0452-82
Company: Invitrogen CD38 PE-Cyanine7 clone: 90 Catalog #: 25-0381-82
Company: Invitrogen GL7 eFluor 45 clone: GL-7 Catalog #: 48-5092-82
Company: Invitrogen CD1d PE clone: 1B1 Catalog #: 12-0011-82
Company: Invitrogen CD21/CD35 PE-Cyanine 7 clone: 8D9 Catalog #: 47-0211-82
Company: Invitrogen TCR β PE clone: H57-597 Catalog #: 12-5961-82
Company: Invitrogen CD69 eFluor 45 clone: H1.2F3 Catalog #: 48-0691-82
Company: Invitrogen CD62L PE-Cyanine7 clone: MEL-14 Catalog #: 25-0621-82
Company: Invitrogen BST2 FITC clone: eBio927 Catalog #: 11-3172-82
Company: Invitrogen SIGLEC-H APC clone: eBio440c Catalog #: 17-0333-82
Company: Invitrogen TNF PE-Cyanine7 clone: MP6-XT22 Catalog #: 25-7321-82
Company: BioLegend CD44 APC-Cyanine7 clone: IM7 Catalog #: 103028
Company: BioLegend CD44 APC clone: IM7 Catalog #: 103012
Company: BioLegend B220 PE clone: RA3-6B2 Catalog #: 103208
Company: BioLegend PD-1 PE-Cyanine7 clone: 29F.1A12 Catalog #: 135216
Company: BioLegend PD-1 APC-Cyanine7 clone: 29F.1A12 Catalog #: 1352224
Company: BioLegend CD138 PE-Cyanine7 clone: 281-2 Catalog #: 142514
Company: BioLegend CD23 PerCP-Cyanine5.5 clone: B3B4 Catalog #: 101618
Company: BioLegend IA/IE APC-Cyanine7 clone: M5/114.15.2 Catalog #: 107628
Company: BioLegend IA/IE APC clone: M5/114.15.2 Catalog #: 107614
Company: BioLegend CXCR3 eFluor 45 clone: CXCR3-173 Catalog #: 126529
Company: BioLegend CD4 PerCP-Cyanine5.5 clone: GK1.5 Catalog #: 100434
Company: BioLegend CD8 BV605 clone: 53-6.7 Catalog #: 100743
Company: BioLegend Ly6G BV605 clone: 1A8 Catalog #: 127612
Company: BioLegend Ly6C FITC clone: HK1.4 Catalog #: 128006
Company: BioLegend F4/80 PE clone: BM8 Catalog #: 123110
Company: BioLegend CD11c APC-Cyanine7 clone: N418 Catalog #: 117352
Company: BioLegend CD45 APC clone: 30-F11 Catalog #: 103112

Company: Pblassay IFNa FITC clone: RMMA-1 Catalog #: 22100-3

For ELISA,
Company: Invitrogen Goat AP-conjugated anti-mouse IgG Catalog #: G-21060

For kidney immunofluorescence,
Company: Thermo Fisher Scientific rabbit anti-mouse IgG Catalog #: A27022
Company: Abcam rat anti-mouse C3 Catalog #: ab11862

For western blotting,
Company: Sigma Anti-Flag (host: Mouse) Catalog #: F1840
Company: Roche Anti-HA (host: Rat) Catalog #: 11867423001
Company: Abcam Calnexin (host: Rabbit) Catalog #: ab213243
Company: Abcam Rab7 (host: Rabbit) Catalog #: ab137029
Company: Abcam Tubulin (host: Mouse) Catalog #: ab78078
Company: Novus PPT1 (host: Rabbit) Catalog #: NBP2-93840
Company: Novus TLR9 (host: Mouse) Catalog #: NBP2-24729
Company: CST anti-biotin, HRP-linked Catalog #: 7075S
Company: Abcam goat anti-mouse IgG H&L (HRP) Catalog #: ab6789
Company: Abcam goat anti-rabbit IgG H&L (HRP) Catalog #: ab6721
Company: Abcam goat anti-rat IgG H&L (HRP) Catalog #: ab97057
Company: Abmart GM130 (host: Rabbit) Catalog #: T55142
Company: CST EEA1 (host: Rabbit) Catalog #: 2411

For Immunoprecipitation,
Company: Sigma anti-Flag matrix Catalog #: A2220
Company: Roche anti-HA matrix Catalog #: 11815016001

Validation

All antibodies used were commercially available and validated by corresponding manufacturers. The following companies have general validation/reproducibility statements. Biolegend (https://www.biolegend.com/en-us/reproducibility). Roche applied science (https://lifescience.roche.com/en_cn/tools-and-resources/documents.html). Sigma aldrich(https://www.sigmaaldrich.com/technical-documents/articles/biology/antibody-standard-validation.html). Thermo Fisher Scientific (https://www.thermofisher.cn/cn/zh/home/life-science/antibodies/invitrogen-antibody-validation.html). Abcam (https://www.abcam.com/primary-antibodies/how-we-validate-our-antibodies).

Company: Invitrogen IgG Catalog #: 13-4013-85 https://www.thermofisher.cn/cn/zh/antibody/product/Rat-anti-Mouse-IgG-H-L-Secondary-Antibody-Monoclonal/13-4013-85
Company: Invitrogen IgD clone: 11-26 Catalog #: 13-5993-82 https://www.thermofisher.cn/cn/zh/antibody/product/IgD-Antibody-clone-11-26c-11-26-Monoclonal/13-5993-82
Company: Invitrogen CD93 clone: AA4.1 Catalog #: 13-5892-82 https://www.thermofisher.cn/cn/zh/antibody/product/CD93-AA4-1-Antibody-clone-AA4-1-Monoclonal/13-5892-82
Company: Invitrogen CD5 clone: 53-7.3 Catalog #: 13-0051-82 https://www.thermofisher.cn/cn/zh/antibody/product/CD5-Antibody-clone-53-7-3-Monoclonal/13-0051-82
Company: Invitrogen CD49b clone: DX5 Catalog #: 13-5971-85 https://www.thermofisher.cn/cn/zh/antibody/product/CD49b-Integrin-alpha-2-Antibody-clone-DX5-Monoclonal/13-5971-82
Company: Invitrogen TCR β clone: H57-597 Catalog #: 13-5961-85 https://www.thermofisher.cn/cn/zh/antibody/product/TCR-beta-Antibody-clone-H57-597-Monoclonal/13-5961-82
Company: Invitrogen CD11b clone: M1170 Catalog #: 13-0112-82 https://www.thermofisher.cn/cn/zh/antibody/product/CD11b-Antibody-clone-M1-70-Monoclonal/13-0112-82
Company: Invitrogen CD24 clone: M1/69 Catalog #: 13-0242-82 https://www.thermofisher.cn/cn/zh/antibody/product/CD24-Antibody-clone-M1-69-Monoclonal/13-0242-82
Company: Invitrogen F4/80 clone: BM8 Catalog #: 13-4801-81 https://www.thermofisher.cn/cn/zh/antibody/product/F4-80-Antibody-clone-BM8-Monoclonal/13-4801-82
Company: BioLegend Ly6G/Ly6C (Gr-1) clone: R86-8C5 Catalog #: 108404 https://www.biolegend.com/ja-jp/products/purified-anti-mouse-ly-6g-ly-6c-gr-1-antibody-462?GroupID=BLG4876
Company: BioLegend CD41 clone: MWReg30 Catalog #: 133930 https://www.biolegend.com/en-us/products/biotin-anti-mouse-cd41-antibody-13059
Company: TONBO CD19 clone: 1D3 Catalog #: 30-0193-U500 no longer available
Company: TONBO TER-119 clone: TER-119 Catalog #: 30-5921-U500 https://cytekbio.com/products/biotin-anti-mouse-ter-119-ter-119?variant=40581229346852
Company: TONBO CD3 clone: 145-2C11 Catalog #: 30-0031-U500 https://cytekbio.com/products/biotin-anti-mouse-cd3e-145-2c11?variant=40581230428196

For flow cytometry ,
Company: Invitrogen CD19 eFluor 450 Clone: 1D3 Catalog #: 48-0193-82 https://www.thermofisher.cn/cn/zh/antibody/product/CD19-Antibody-clone-eBio1D3-1D3-Monoclonal/48-0193-82
Company: Invitrogen CD3e APC clone: 17A2 Catalog #: 17-0032-82 https://www.thermofisher.cn/cn/zh/antibody/product/CD3-Antibody-clone-17A2-Monoclonal/17-0032-82
Company: Invitrogen CD11b PerCP-Cyanine5.5 clone: M1/70 Catalog #: 45-0112-82 https://www.thermofisher.cn/cn/zh/antibody/product/CD11b-Antibody-clone-M1-70-Monoclonal/45-0112-82
Company: Invitrogen IgD APC-Cyanine7 clone: 11-26c Catalog #: 47-5993-82 https://www.thermofisher.cn/cn/zh/antibody/product/

IgD-Antibody-clone-11-26c-11-26-Monoclonal/25-5993-82
Company: Invitrogen IgM PE clone: II/41 Catalog #: 12-5790-82 https://www.thermofisher.cn/cn/zh/antibody/product/IgM-Antibody-clone-II-41-Monoclonal/12-5790-82
Company: Invitrogen B220 FITC clone: RA3-6B2 Catalog #: 11-0452-82 https://www.thermofisher.cn/cn/zh/antibody/product/CD45R-B220-Antibody-clone-RA3-6B2-Monoclonal/11-0452-82
Company: Invitrogen CD38 PE-Cyanine7 clone: 90 Catalog #: 25-0381-82 https://www.thermofisher.cn/cn/zh/antibody/product/CD38-Antibody-clone-90-Monoclonal/25-0381-82
Company: Invitrogen GL7 eFluor 45 clone: GL-7 Catalog #: 48-5092-82 https://www.thermofisher.cn/cn/zh/antibody/product/GL7-Antibody-clone-GL-7-GL7-Monoclonal/48-5902-82
Company: Invitrogen CD1d PE clone: 1B1 Catalog #: 12-0011-82 https://www.thermofisher.cn/cn/zh/antibody/product/CD1d-Antibody-clone-1B1-Monoclonal/12-0011-82
Company: Invitrogen CD21/CD35 PE-Cyanine 7 clone: 8D9 Catalog #: 47-0211-82 https://www.thermofisher.cn/cn/zh/antibody/product/CD21-CD35-Antibody-clone-eBio8D9-8D9-Monoclonal/25-0211-82
Company: Invitrogen TCR β PE clone: H57-597 Catalog #: 12-5961-82 https://www.thermofisher.cn/cn/zh/antibody/product/TCR-beta-Antibody-clone-H57-597-Monoclonal/12-5961-82
Company: Invitrogen CD69 eFluor 45 clone: H1.2F3 Catalog #: 48-0691-82 https://www.thermofisher.cn/cn/zh/antibody/product/CD69-Antibody-clone-H1-2F3-Monoclonal/48-0691-82
Company: Invitrogen CD62L PE-Cyanine7 clone: MEL-14 Catalog #: 25-0621-82 https://www.thermofisher.cn/cn/zh/antibody/product/CD62L-L-Selectin-Antibody-clone-MEL-14-Monoclonal/25-0621-82
Company: Invitrogen BST2 FITC clone: eBio927 Catalog #: 11-3172-82 https://www.thermofisher.cn/cn/zh/antibody/product/CD317-BST2-PDCA-1-Antibody-clone-eBio927-Monoclonal/11-3172-82
Company: Invitrogen SIGLEC-H APC clone: eBio440c Catalog #: 17-0333-82 https://www.thermofisher.cn/cn/zh/antibody/product/SIGLEC-H-Antibody-clone-eBio440c-Monoclonal/17-0333-82
Company: Invitrogen TNF PE-Cyanine7 clone: MP6-XT22 Catalog #: 25-7321-82 https://www.thermofisher.cn/cn/zh/antibody/product/TNF-alpha-Antibody-clone-MP6-XT22-Monoclonal/25-7321-82
Company: BioLegend CD44 APC-Cyanine7 clone: IM7 Catalog #: 103028 https://www.biolegend.com/en-us/products/apc-cyanine7-anti-mouse-human-cd44-antibody-3933?GroupID=BLG10248
Company: BioLegend CD44 APC clone: IM7 Catalog #: 103012 https://www.biolegend.com/ja-jp/products/apc-anti-mouse-human-cd44-antibody-312
Company: BioLegend B220 PE clone: RA3-6B2 Catalog #: 103208 https://www.biolegend.com/de-de/products/pe-anti-mouse-human-cd45r-b220-antibody-447
Company: BioLegend PD-1 PE-Cyanine7 clone: 29F.1A12 Catalog #: 135216 https://www.biolegend.com/en-us/products/pe-cyanine7-anti-mouse-cd279-pd-1-antibody-7005?GroupID=BLG7928
Company: BioLegend PD-1 APC-Cyanine7 clone: 29F.1A12 Catalog #: 1352224 https://www.biolegend.com/en-us/products/apc-cyanine7-anti-mouse-cd279-pd-1-antibody-9742
Company: BioLegend CD138 PE-Cyanine7 clone: 281-2 Catalog #: 142514 https://www.biolegend.com/nl-nl/products/pe-cyanine7-anti-mouse-cd138-syndecan-1-antibody-8601
Company: BioLegend CD23 PerCP-Cyanine5.5 clone: B3B4 Catalog #: 101618 https://www.biolegend.com/en-us/products/percp-cyanine5-5-anti-mouse-cd23-antibody-9118?GroupID=BLG1926
Company: BioLegend IA/IE APC-Cyanine7 clone: M5/114.15.2 Catalog #: 107628 https://www.biolegend.com/en-us/products/apc-cyanine7-anti-mouse-i-a-i-e-antibody-5966?GroupID=BLG11931
Company: BioLegend IA/IE APC clone: M5/114.15.2 Catalog #: 107614 https://www.biolegend.com/nl-be/products/apc-anti-mouse-i-a-i-e-antibody-2488
Company: BioLegend CXCR3 eFluor 45 clone: CXCR3-173 Catalog #: 126529 https://www.biolegend.com/en-us/products/brilliant-violet-421-anti-mouse-cd183-cxcr3-antibody-7159?GroupID=BLG10642
Company: BioLegend CD4 PerCP-Cyanine5.5 clone: GK1.5 Catalog #: 100434 https://www.biolegend.com/en-gb/products/percp-cyanine5-5-anti-mouse-cd4-antibody-4220?GroupID=BLG4745
Company: BioLegend CD8 BV605 clone: 53-6.7 Catalog #: 100743 https://www.biolegend.com/nl-nl/products/brilliant-violet-605-anti-mouse-cd8a-antibody-7636
Company: BioLegend Ly6G BV605 clone: 1A8 Catalog #: 127612 https://www.biolegend.com/fr-fr/products/brilliant-violet-605-anti-mouse-ly-6g-antibody-12244
Company: BioLegend Ly6C FITC clone: HK1.4 Catalog #: 128006 https://www.biolegend.com/de-at/products/fitc-anti-mouse-ly-6c-antibody-4896
Company: BioLegend F4/80 PE clone: BM8 Catalog #: 123110 https://www.biolegend.com/en-us/products/pe-anti-mouse-f4-80-antibody-4068?GroupID=BLG5319
Company: BioLegend CD11c APC-Cyanine7 clone: N418 Catalog #: 117352 https://www.biolegend.com/fr-ch/products/apc-cyanine7-anti-mouse-cd11c-antibody-3931
Company: BioLegend CD45 APC clone: 30-F11 Catalog #: 103112 https://www.biolegend.com/de-at/products/apc-anti-mouse-cd45-antibody-97
Company: Pblassay IFNa FITC clone: RMMA-1 Catalog #: 22100-3 https://www.pblassaysci.com/antibodies/fitc-conjugated-anti-mouse-ifn-alpha-antibody-clone-rmma-1-mab-221003

For ELISA,
Company: Invitrogen Goat AP-conjugated anti-mouse IgG Catalog #: G-21060 https://www.thermofisher.cn/cn/zh/antibody/product/Goat-anti-Mouse-IgG-H-L-Cross-Adsorbed-Secondary-Antibody-Polyclonal/G-21060

For kidney immunofluorescence,
Company: Thermo Fisher Scientific rabbit anti-mouse IgG Catalog #: A27022 https://www.thermofisher.cn/cn/zh/antibody/product/Rabbit-anti-Mouse-IgG-H-L-Secondary-Antibody-Recombinant-Polyclonal/A27022
Company: Abcam rat anti-mouse C3 Catalog #: ab11862 https://www.abcam.com/products/primary-antibodies/c3c3b-antibody-11h9-ab11862.html

For western blotting,
Company: Sigma Anti-Flag (host: Mouse) Catalog #: F1840 https://www.sigmaaldrich.com/HK/zh/product/sigma/f1804
Company: Roche Anti-HA (host: Rat) Catalog #: 11867423001 https://www.sigmaaldrich.com/HK/zh/product/roche/roahaha
Company: Abcam Calnexin (host: Rabbit) Catalog #: ab213243 https://www.abcam.com/products/primary-antibodies/calnexin-antibody-epr21205-ab213243.html
Company: Abcam Rab7 (host: Rabbit) Catalog #: ab137029 https://www.abcam.com/products/primary-antibodies/rab7-antibody-epr7589-ab137029.html
Company: Abcam Tubulin (host: Mouse) Catalog #: ab78078 https://www.abcam.com/products/primary-antibodies/beta-iii-tubulin-antibody-2g10-neuronal-marker-ab78078.html
Company: Novus PPT1 (host: Rabbit) Catalog #: NBP2-93840 https://www.novusbio.com/products/ppt1-antibody-oti1f10_nbp2-45388
Company: Novus TLR9 (host: Mouse) Catalog #: NBP2-24729 https://www.novusbio.com/products/tlr9-antibody-26c5932_nbp2-24729
Company: CST anti-biotin, HRP-linked Catalog #: 7075S https://www.cellsignal.com/products/secondary-antibodies/anti-biotin-hrp-linked-antibody/7075
Company: Abcam goat anti-mouse IgG H&L (HRP) Catalog #: ab6789 https://www.abcam.com/products/secondary-antibodies/goat-mouse-igg-hl-hrp-ab6789.html
Company: Abcam goat anti-rabbit IgG H&L (HRP) Catalog #: ab6721 https://www.abcam.com/products/secondary-antibodies/goat-rabbit-igg-hl-hrp-ab6721.html
Company: Abcam goat anti-rat IgG H&L (HRP) Catalog #: ab97057 https://www.abcam.com/products/secondary-antibodies/goat-rat-igg-hl-hrp-ab97057.html
Company: Abmart GM130 (host: Rabbit) Catalog #: T55142 http://www.ab-mart.com.cn/page.aspx?node=%2077%20&id=%201436
Company: CST EEA1 (host: Rabbit) Catalog #: 2411 https://www.cellsignal.com/products/primary-antibodies/eea1-c45b10-rabbit-mab/3288?_requestid=4824795

For Immunoprecipitation,
Company: Sigma anti-Flag matrix Catalog #: A2220 https://www.sigmaaldrich.com/HK/zh/product/sigma/a2220
Company: Roche anti-HA matrix Catalog #: 11815016001 https://www.sigmaaldrich.cn/CN/zh/product/roche/11815016001

Eukaryotic cell lines

Policy information about cell lines and Sex and Gender in Research

Cell line source(s)
293T were purchased from ATCC (CRL-3216), Flt3L-B16 cell line is generated from ATCC-purchased B16 (ATCC, CRL-6475).L929 is purchase from ATCC (CRL-6364), RAW264.7 macrophage cell lines is purchase from ATCC (TIB-71), Human monocytic THP-1 cells is purchase from ATCC (TIB-202)

Authentication
Authentication of 293T ,B16, L929 is provided by ATCC with STR profiling. The complete authentication information is provided at: 293T: https://www.atcc.org/products/all/CRL-3216.aspx; B16: https://www.atcc.org/products/all/CRL-6475.aspx; L929: https://www.atcc.org/products/all/CRL-6364.aspx; RAW264.7: https://www.atcc.org/products/tib-71.aspx; THP-1: https://www.atcc.org/products/tib-202.aspx. Cell lines were authenticated via morphology, functional assays.

Mycoplasma contamination
The cell line was confirmed to be negative for mycoplasma contamination by PCR.

Commonly misidentified lines
(See ICLAC register)
No commonly misidentified cell lines were used.

Animals and other research organisms

Policy information about studies involving animals; ARRIVE guidelines recommended for reporting animal research, and Sex and Gender in Research

Laboratory animals
C57BL/6J (WT), B6.SJL-PtprcaPepcb/BoyJ (Strain #: 002014, CD45.1+), B6.129S6-Ppt1tm1Hof/SopJ, (Strain #: 006566, Ppt1−/−), B6.Cg-Sle1NZM2410/AegYaa/DcrJ (Strain #: 021569, B6. Sle1yaa) and C57BL/6-Tg (CLEC4C-HBEGF)956Cln/J (Strain #: 014176, BDCA2-DTR) mice were purchased from the Jackson Laboratory. B6.129S6-Ppt1tm1Hof/SopJ (Ppt1−/−) mice were back-crossed for ≥10 generations with C57BL/6J. Ppt1-/- B6.Sle1yaa, and Ppt1+/+ B6.Sle1yaa mice were generated by crossing B6.Cg-Sle1NZM2410/AegYaa/DcrJ (B6.Sle1yaa) males with backcrossed B6.129S6-Ppt1tm1Hof/SopJ (Ppt1−/−) females for two generations. All mice used are sex matched with no bias for female or male, except B6.Sle1yaa related strains, at the age -matched range of 8-16 weeks. The mice are housed in 12 hours/12 hours light-dark cycle, ambient temperature of 18-22 degrees, and 50-60% humidity.

Wild animals
This study did not involve the use of wild animals.

Reporting on sex
B6.Sle1yaa related strains (B6.Sle1yaa, Ppt1-/- B6.Sle1yaa, and Ppt1+/+ B6.Sle1yaa) are all male mice presented on Fig1; Fig2; Supplementary Fig1; Supplementary Fig2. Because B6.Sle1yaa mice only can be developed into lupus-like phenotype with Yaa-containing Y chromosome.

Field-collected samples
The study did not involve samples collected from the field.

Ethics oversight

All mice protocols were followed according to the institutional guidelines and protocols approved by the Animal Ethics Committee of Sun Yat-sen University.

Note that full information on the approval of the study protocol must also be provided in the manuscript.

Flow Cytometry

Plots

Confirm that:

- [ ] The axis labels state the marker and fluorochrome used (e.g. CD4-FITC).
- [x] The axis scales are clearly visible. Include numbers along axes only for bottom left plot of group (a 'group' is an analysis of identical markers).
- [x] All plots are contour plots with outliers or pseudocolor plots.
- [x] A numerical value for number of cells or percentage (with statistics) is provided.

Methodology

Sample preparation

Cells were blocked with Fc blocker(CD16/32), and stained for specific surface markers. For intracellular staining, cells were fixed and permeabilized and stained for intracellular cytokines by fixation/permeabilization kit (eBiosciences).

Instrument

Flow cytometry data were collected by CytoFLEX (Beckman Coulter) and FACSAria III (BD Biosciences) for cell sorting.

Software

CytoFLEX (Beckman Coulter) software for data acquisition and analyzed using FlowJo software version X (Tree Star). Data was graphed using Prism 8 (Graphpad).

Cell population abundance

After sorting the purity was determined to be >95%.

Gating strategy

All antibodies were titrated on murine splenic cells to determine optional concentrations for the separation of positive or negative populations. All population markers were on bifurcated markers and clearly defined positive from negative.

All immune cells are gated with FSC-A/SSC-A for a live gate. Then a viability dye (FVD506) is used to exclude dead cells. Next, FSC-A/FSC-H is used to exclude doublets. After these gating, cells are specifically gated accordingly below:

a. Gating strategy (CD11b+) to identify myeloid cells presented on Fig. 1F; Fig2G.

b. Gating strategies (TCR β+ CD4+ CD44+ CD62L-) to activated CD4+ T cells presented on Fig. 1G; Fig. 2H.

c. Gating strategies (TCR β+ CD8+ CD44+ CD62L-) to activated CD8+ T cells presented on Fig. 1H; Fig. 2I.

d. Gating strategies (CD3- CD19+ MHCII+ CXCR3+) to antibody-forming B cells presented on Fig. 1I; Fig. 2J.

e. Gating strategies (CD3+) to CD3+ T cells and (CD19+) to B cells presented on Supplementary Fig. 1A; Supplementary Fig. 2A.

f. Gating strategies (B220+ IgD- CD38- GL-7+) to germinal center B cells presented on Supplementary Fig. 1B; Supplementary Fig. 2B.

g. Gating strategies (B220- CD19- CD138+) to plasma B cells presented on Supplementary Fig. 1C; Supplementary Fig. 2C.

h. Gating strategies (CD3- CD19+ IgM+ IgD-) to immature B cells and (CD3- CD19+ IgM- IgD+) to mature B cells presented on Supplementary Fig. 1D; Supplementary Fig. 2D.

i. Gating strategies (B220+ CD11c+ SiglecH+) to identify pDCs or sort BM pDCs presented on Fig. 3C, 3F; Supplementary Fig. 3D-F, 3I, 3K.

j. Gating strategies (B220+ CD11c+ SiglecH+) to Flt3l-pDCs presented on Fig. 3B.

k. Gating strategies (CD11b+ F4/80+) to identify or sort peritoneal cavity macrophages or BMDMs presented on Fig 3E-F.

l. Gating strategies (CD19+) to identify splenic B cells presented on Fig. 3E, 3F; Supplementary Fig. 3H, 3J, 7D-E.

- [x] Tick this box to confirm that a figure exemplifying the gating strategy is provided in the Supplementary Information.
